# Alpha-Synuclein Disrupted Dopamine Homeostasis Leads to Dopaminergic Neuron Degeneration in *Caenorhabditis elegans*

**DOI:** 10.1371/journal.pone.0009312

**Published:** 2010-02-19

**Authors:** Pengxiu Cao, Yiyuan Yuan, Elizabeth A. Pehek, Alex R. Moise, Ying Huang, Krzysztof Palczewski, Zhaoyang Feng

**Affiliations:** 1 Department of Pharmacology, School of Medicine, Case Western Reserve University, Cleveland, Ohio, United States of America; 2 Departments of Psychiatry and Neurosciences, School of Medicine, Case Western Reserve University, Cleveland, Ohio, United States of America; 3 Department of Veterans Affairs, Louis Stokes Cleveland Veterans Affairs Medical Center, Cleveland, Ohio, United States of America; 4 Department of Physiology, College of Medicine, Xi'an Jiaotong University, Xi'an, Shaanxi, China; University of Nebraska, United States of America

## Abstract

Disruption of dopamine homeostasis may lead to dopaminergic neuron degeneration, a proposed explanation for the specific vulnerability of dopaminergic neurons in Parkinson's disease. While expression of human α-synuclein in *C. elegans* results in dopaminergic neuron degeneration, the effects of α-synuclein on dopamine homeostasis and its contribution to dopaminergic neuron degeneration in *C. elegans* have not been reported. Here, we examined the effects of α-synuclein overexpression on worm dopamine homeostasis. We found that α-synuclein expression results in upregulation of dopamine synthesis and content, and redistribution of dopaminergic synaptic vesicles, which significantly contribute to dopaminergic neuron degeneration. These results provide *in vivo* evidence supporting a critical role for dopamine homeostasis in supporting dopaminergic neuron integrity.

## Introduction

Abnormal dopamine (DA) metabolism, which produces reactive oxygen species (ROS), may lead to dopaminergic (DAergic) neuron degeneration and has been proposed to be related to the pathogenesis of Parkinson's Disease (PD) [1]–[6]. For example, overexpression of tyrosine hydroxylase (TH) in primary neuronal cultures of *Drosophila* embryos induces cellular degeneration [1] and vesicular monoamine transporter (VMAT) loss-of-function mice show nigrostriatal neurogdegeneration [2].

Some *in vitro* or *ex vivo* evidence also suggests a connection between dopamine homeostasis and α-synuclein, the central player of PD pathology [3]–[11]. Thus, expression of pathogenic α-synuclein mutants enhances cytosolic catecholamine levels in human mesencephalic cells, PC12 cells and mouse chromaffin cells [12], [13]. Moreover, genetic disruption of vesicular dopamine storage induces age-dependent alterations in the nigrostriatal dopamine system and progressive nigral cell loss in α-synuclein positive, but not in α-synuclein negative mice [2]. Reduction of cytosolic dopamine content either genetically or pharmacologically prevents hαSyn-mediated neuronal degeneration *in vitro* [1]. It also has been suggested that α-synuclein overexpression disrupts vesicular pH, leading to the increased cytosolic catechol species [13].

Genetic model organisms such as yeast, *Drosophila* and *C. elegans* are valuable surrogates for the study of certain aspects of neurodegenerative diseases, including investigations of α-synuclein toxicity [5], [14]–[22]. For example, genes involved in protein trafficking have recently been identified to be involved in α-synuclein toxicity, leading to the hypothesis that α-synuclein mediated altered intracellular trafficking regulates dopamine homeostasis [5].

Expression of human α-synuclein (hαSyn) in DAergic neurons of *C. elegans* results in their degeneration [21], [22]. Yet, the effects of hαSyn expression on dopamine homeostasis have not been addressed in this useful organism. Here, we used hαSyn-expressing *C. elegans* lines to examine the toxic effects of hαSyn on dopamine homeostasis and its contribution to hαSyn-mediated DAergic neuron degeneration.

## Results

### hαSyn Expression Induces DAergic Neuron Degeneration

We first characterized the expression of *dat-1* promoter-driven hαSyn by using immunohistochemistry and confocal microscopy. Positive hαSyn immunostaining was found exclusively in DAergic neurons, marked with *dat-1* promoter-driven DsRed, demonstrating the specificity of hαSyn expression in our transgenic lines (Figure S1).

**Figure 1:**
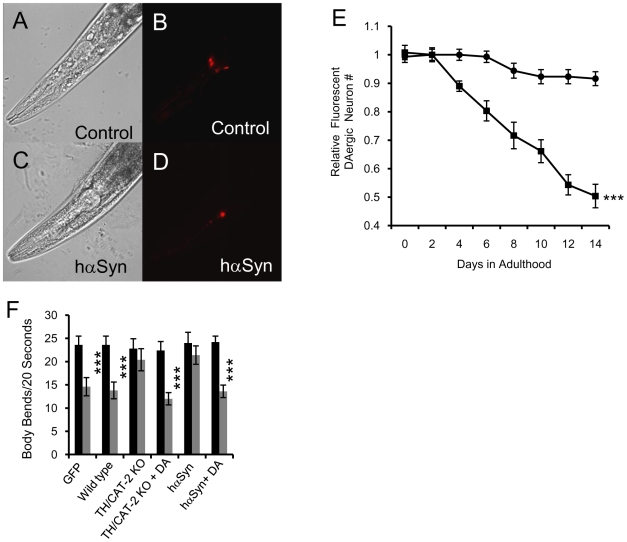
hαSyn expression leads to DAergic neuron degeneration. A–D, Confocal images of living day 10 adult control (AB) or hαSyn-expressing (CD) worms with DAergic neuron specific expression of DsRed. A and C, Bright field; B and D, DsRed. E, Number of fluorescent DAergic neurons in hαSyn-expressing (squares) and control (circles) lines. ***, p<0.005 (two-way ANOVA). Error bars represent the SEM (standard error of the mean) for three independent experiments. In each experiment, the n of each sample varied from 20 to 50. F, Basal slowing response of day 2 adult worms. TH/CAT-2 KO or *cat-2* is a TH/CAT-2 knockout (KO) worm line. GFP indicates a wild type line expressing GFP in DAergic neurons. Food response experiments were conducted with (grey bars) or without (black bars) food. ***, p<0.0001 (t-test); n varied from 6 to 20. Error bars indicate SEM.

Previous efforts to express wild type or pathogenic hαSyn in worms led to loss of the fluorescent DAergic neuron marker due to degeneration of DAergic neurons [5], [20]. Consistent with these reports, our hαSyn-expressing line, but not the control line, displayed an age-related progressive decline in the number of fluorescent DAergic neurons (Figure 1A–E). Another hαSyn-expressing line also exhibited a similar decline in the number of fluorescent DAergic neurons (data not shown). This conclusion was further confirmed by TH immunostaining experiments (Figure S2) and similar experiments where both a non-functional CAT-2/TH::GFP fusion protein [23], [24] and DsRed were used as DAergic neuron markers (Figure S3).

We next investigated the effect of hαSyn expression on the function of worm DAergic neurons by measuring the basal slowing response, a food-sensing behavior regulated by dopamine neurotransmission [25]. The worm basal slowing response was used to assess the effect of hαSyn expression on the function of DAergic neurons [21]. As found in *cat-2*, a knockout mutant of worm TH, hαSyn-expressing worms had an impaired basal slowing response, which returned to control levels in the presence of 0.5 mM exogenous dopamine (Figure 1F). Thus, animals of the hαSyn expressing line were functionally deficient in dopamine.

Consistent with our hαSyn expression pattern, the enhanced slowing response, a food response behavior regulated by serotonin neurotransmission [25], was not affected in hαSyn expressing animals (Figure S4).

Taken together, these results lead us to conclude that hαSyn expression induces degeneration of DAergic neurons in our hαSyn expressing lines, similar to previous reports.

### hαSyn Expression Induces a Motor Capacity Deficit

**Figure 2:**
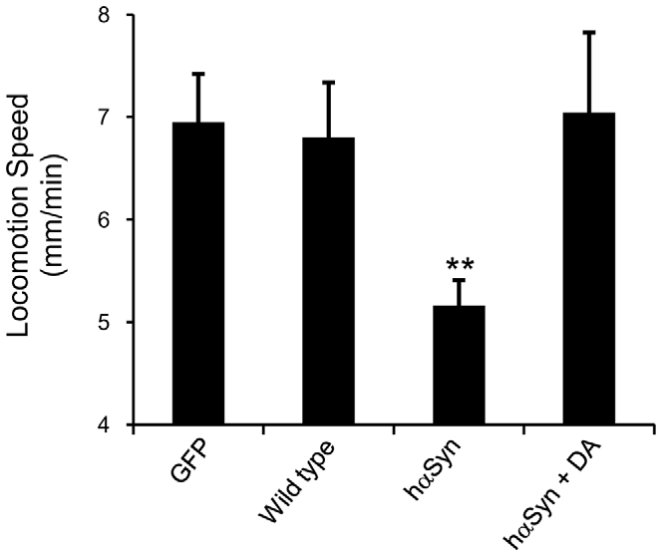
hαSyn expression leads to a motor deficit. Locomotion speed was quantified in day 2 adult worms. **: p<0.01 (one way ANOVA with Dunnet's post-hoc test). n varies 10 to 15. Error bars indicate SEM. This deficit was not observed after addition of 1 mM DA.

We next quantified the effect of hαSyn expression on worm motor capacity, which had not been assessed previously in worms specifically expressing hαSyn in DAergic neurons [5], [21]. In general, there are two methods to access motor capacity in worms: body bending frequency and centroid velocity [25]–[28]. Body bending frequency is the number of sinusoidal waves made by a worm during a given time period, while centroid velocity quantifies the physical displacement of a worm's centroid. Body bending frequency can be uncoupled from centroid displacement by genetic mutations and ageing [26], [29]. We observed that L4 and day 1 adult worms exhibit similar body bending frequencies, although adult worms move much faster than L4 worms, as quantified by their centroid velocity (Cao and Feng, unpublished data). Because the centroid velocity of worm locomotion has been utilized to quantify age-related changes in motor capacity and provides more sensitive and reliable quantification of worm motor activity [26], [27], this parameter was selected to address the effect of hαSyn expression on the worm motor system. Indeed, hαSyn expressing worms exhibited a deficit in motor activity that was restored by adding 1 mM dopamine (Figure 2), a finding consistent with observations in a *Drosophila* PD model [17].

### hαSyn Expression Results in Altered Dopamine Metabolism

**Figure 3:**
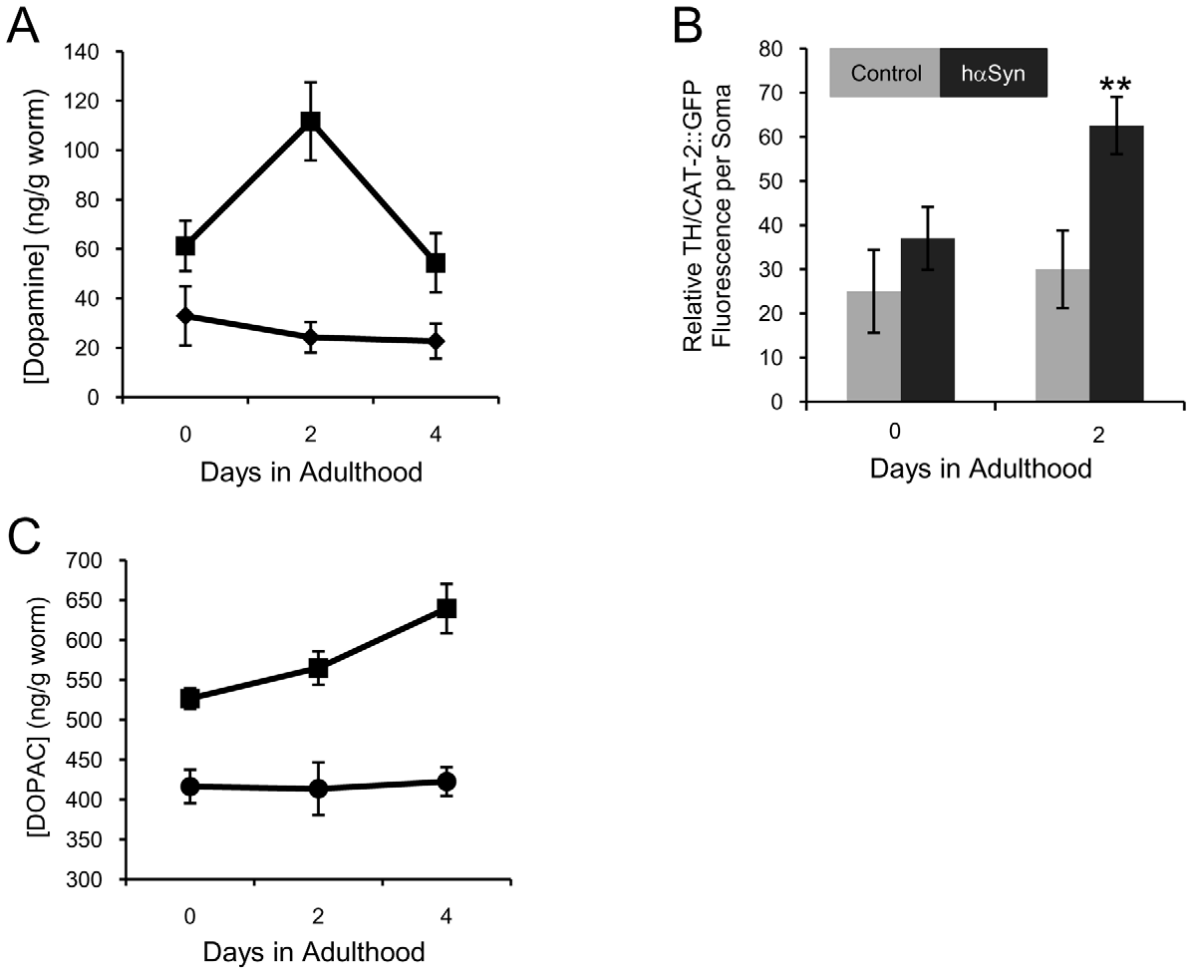
hαSyn expression leads to altered dopamine metabolism. A, Quantification of dopamine content in worms with (squares) or without (diamonds) hαSyn expression is shown as a function of age. Error bars represent the SEM of 3 independent experiments. Each experiment was done with ∼200 worms per sample. B, Quantification of CAT-2::GFP florescence in DAergic neurons of EM641 worms (a worm line expressing a non-functional CAT-2::GFP) either with (black bars) or without (gray bars) hαSyn expression. ** p<0.01 (t-test). n varied from 9 to 12. Error bar, SEM. C, Quantification of DOPAC content in hαSyn expressing (squares) or control (circles) worms. Error bars represent the SEM of 3 independent experiments. Each experiment was done with ∼400 worms per sample.

Despite their functional deficiency in dopamine neurotransmission, hαSyn expressing worms surprisingly exhibited a remarkable upregulation of dopamine content from L4 to day 4 in adulthood (Figure 3A), as measured by liquid chromatography-mass spectrometry (LC-MS). We obtained similar results and reached the same conclusion (data not shown) by using conventional high performance liquid chromatography (HPLC) as well. Consistently, the fluorescence intensity of a non-functional TH/CAT-2::GFP fusion protein [23], [24] in day 2 adult hαSyn expressing worms was significantly elevated (Figure 3B).

Abnormal dopamine metabolism may produce cytotoxic molecules such as hydrogen peroxide, superoxide radicals and dopamine-quinone through two pathways, namely auto-oxidation and deamination by monoamine oxidase (MO). Dopamine deamination also yields 3,4-dihydroxyphenylacetic acid (DOPAC), a non-toxic metabolite that can be used to monitor dopamine deamination-specific oxidative stress [12], [30].

We found that hαSyn-expressing worms displayed an age-related accumulation of DOPAC leading to a significantly higher DOPAC content than control worms (Figure 3C), thereby providing evidence for an hαSyn-mediated disruption of dopamine metabolism. Dopamine-quinone was not detected in any worms (data not shown), possibly because dopamine auto-oxidation is negligible *in vivo*. This quinone can be oxidized to several other species [30] or become adducted to glutathione and/or thiol groups of native proteins [31]. Nevertheless, we conclude that hαSyn expression alters dopamine metabolism in worms.

### hαSyn Expression Redistributes Dopamine Synaptic Vesicles

**Figure 4:**
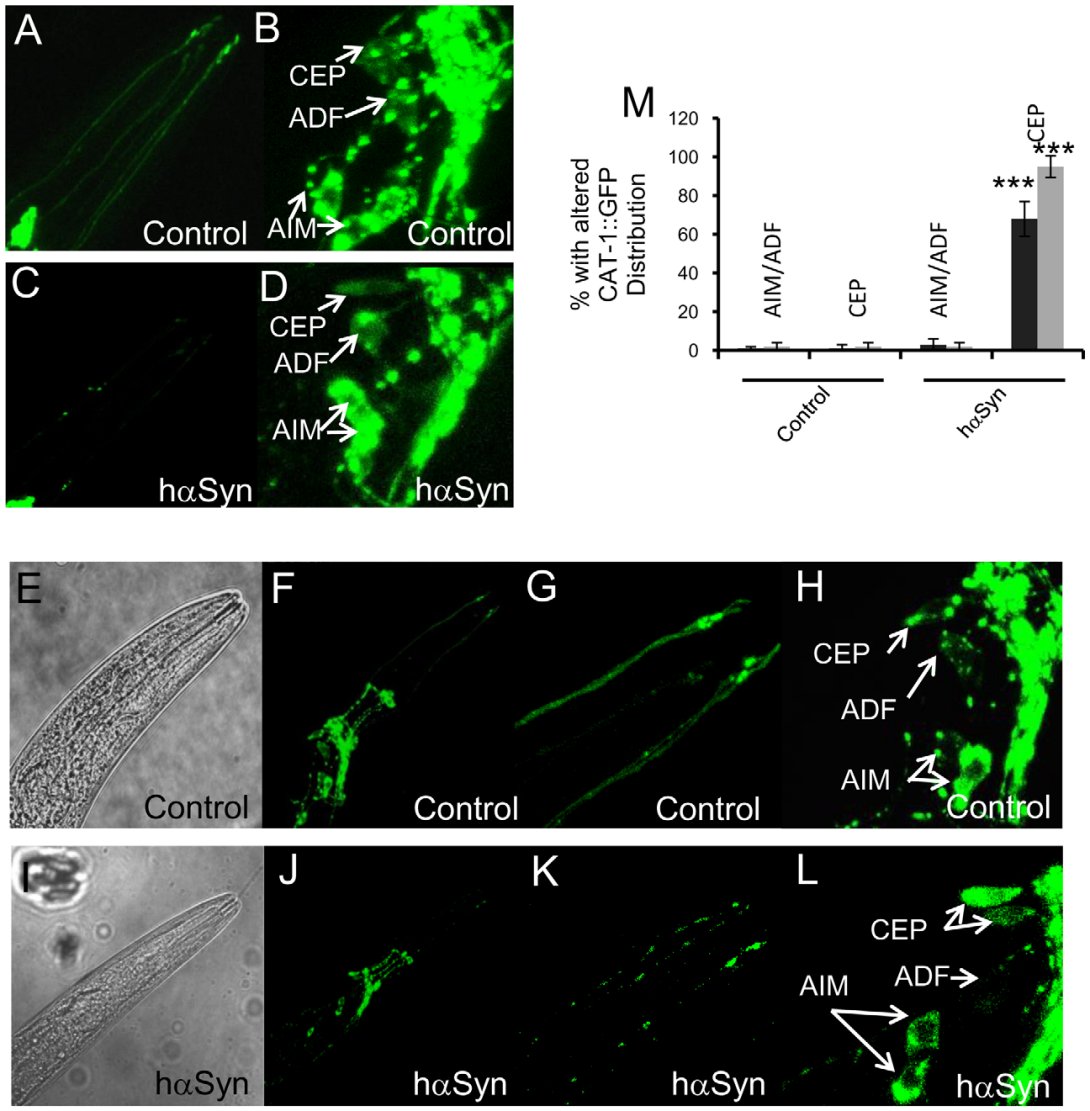
hαSyn expression disrupts dopamine synaptic vesicle distribution. A–L, Typical confocal laser scanning VMAT/CAT-1::GFP (A–D, F–H, J–L) or bright field (E, I) images of living L2 (A–D) or L4 (E–L) nuls26 (a worm line expressing VMAT-CAT-1::GFP) worms expressing (C–D, I–L) or not expressing (A–B, E–H) hαSyn. A–D, are GFP images that show DAergic (CEP, specifically) dendrites (A, C) or DAergic/serotonergic somas (B, D) of L2 worms. G and K are magnified areas of F and J, respectively, that show DAergic dendrites (CEP) of L4 worms. H and L, are magnified areas of F and J, respectively, that show DAergic and serotonergic somas of L4 worms. M, Quantification of CAT-1::GFP redistribution in DAergic neurons (CEP) and serotonergic neurons (AIM and ADF) of L2 (black bars, n = 5) and L4 worms (gray bars, n = 8). ***: p<0.0001 (t-test). Error bars indicate SEM.

Dopamine is loaded into synaptic vesicles by a VMAT and pathogenic α-synuclein impairs dopamine storage in mammalian cell lines [32], [33]. To further investigate whether hαSyn expression affects dopamine homeostasis in worms, we crossed our hαSyn expressing line with a worm line expressing CAT-1::GFP [34]. CAT-1 is the sole worm homolog of VMAT. In worms expressing only VMAT/CAT-1::GFP but not hαSyn, the observed VMAT/CAT-1::GFP expression pattern of DAergic neurites was continuously linear with a few bright spots at both L2 (Figure 4A) and L4 (Figure 4E–G) stages, a finding consistent with previous reports [34]–[36]. In contrast, many bright VMAT/CAT-1::GFP spots appeared in the remarkably weakened linear fluorescent DAergic neurites of hαSyn expressing L2 worms (Figure 4C). Such an hαSyn mediated alteration of VMAT/CAT-1::GFP distribution further developed, and VMAT/CAT-1::GFP fluorescence of DAergic neurites was only located in discrete punctate spots without visible lines in L4 worms (Figure 4I–M), which was prior to the obvious start of DAergic neuron degeneration in this worm variant.

Also consistent with previous reports [34]–[36], VMAT/CAT-1::GFP in DAergic somas of control worms was excluded from the nucleus and formed a punctate pattern in both DAergic and serotonergic neuron somas (Figure 4B and H). hαSyn expression disrupted this pattern of VMAT/CAT-1::GFP expression exclusively in DAergic but not serotonergic neurons as early as L2 (Figure 4D, L–M). From this evidence, we conclude that hαSyn expression causes dopamine synaptic vesicle maldistribution.

### Disruption of hαSyn-Mediated Dopamine Homeostasis Contributes to DAergic Neuron Degeneration

**Figure 5:**
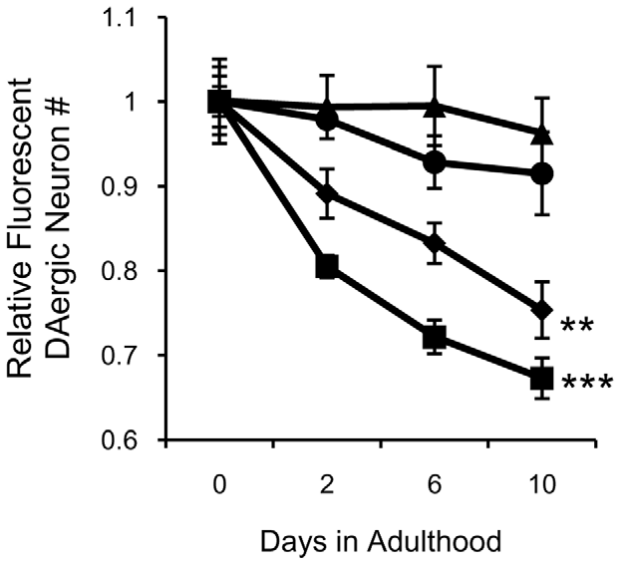
Knockout of TH protects DAergic neurons from hαSyn expression toxicity. A. Quantification of fluorescent DAergic neurons in hαSyn expressing wild type (squares) or hαSyn expressing TH/CAT-2 KO (diamonds) worms and in control wild type (triangles) or TH/CAT-2 KO (circles) worms. Error bars represent the SEM of three independent experiments. **, p<0.01; ***, p<0.005 (Two-way ANOVA). In each experiment, n of each sample varied from 10 to 30.

The next step was to determine whether hαSyn-mediated disruption of dopamine homeostasis contributes to DAergic neuron degeneration in worms. In rodents, exogenous expression of DAT-1, a dopamine transporter, leads to neuronal degeneration. In worms, overexpression of TH/CAT-2 produces DAergic neuron (CEP) abnormalities [22]. Here, we found that hαSyn induced DAergic neuron degeneration more slowly in worms with a *cat-2* mutant background (Figure 5), indicating that hαSyn-mediated DAergic neuron degeneration is related to dopamine homeostasis.

**Figure 6:**
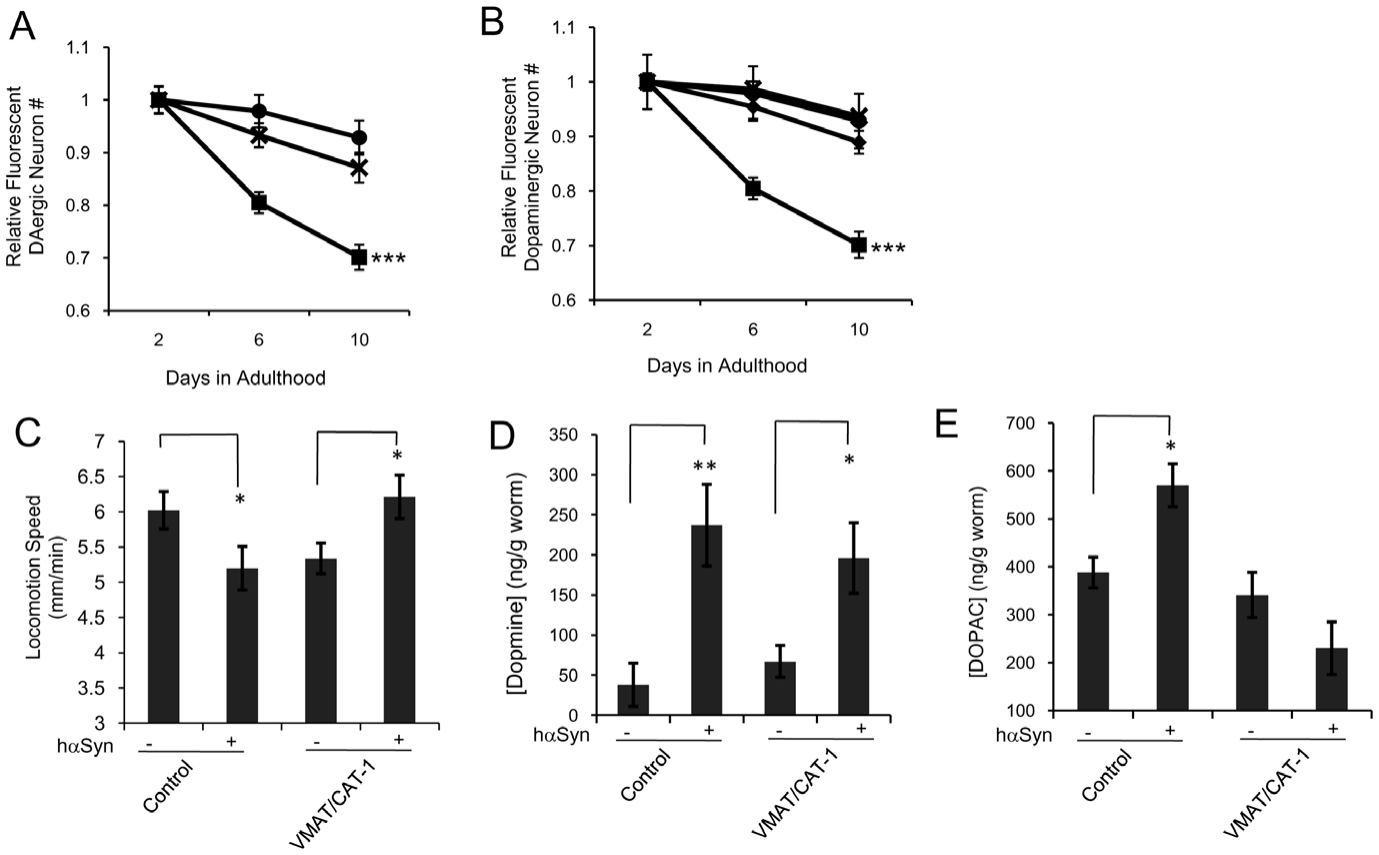
Overexpression of VMAT protects DAergic neurons from hαSyn toxicity. A, Quantification of fluorescent DAergic neurons of wild type (circles), VMAT/CAT-1 KO (crosses), and hαSyn expressing wild type (squares) worms. Error bars represent the SEM of three independent experiments. In each experiment, n of each sample varied from 10 to 25. ***, p<0.005 (Two-way ANOVA). B, Quantification of fluorescent DAergic neurons in wild type (circles), hαSyn expressing wild type(squares), VMAT/CAT-1 overexpressing (diamonds), and hαSyn expressing VMAT/CAT-1 overexpressing (crosses) worms. Error bars represent the SEM of three independent experiments. In each experiment, n of each sample varied from 10 to 30. ***, p<0.005 (Two-way ANOVA). C, Locomotion speed of day 2 adult worms of control and VMAT/CAT-1 overexpressing lines with or without hαSyn expression. *: p<0.01 (t-test). n≥10. Error bars:SEM. D. Quantification of [dopamine] in day 2 adult worms of control and VMAT/CAT-1 overexpressing lines with or without hαSyn expression. Error bars represent the SEM of three independent experiments. In each experiment, the number of worms per sample was about 200. *: p<0.01 (t-test), **: p<0.005 (t-test). E. Quantification of [DOPAC] in day 2 adult worms of control and VMAT/CAT-1 overexpressing lines with or without hαSyn expression. Error bars represent the SEM of three independent experiments. In each experiment, the number of worms per sample was about 200. *: p<0.01 (t-test).

Dopamine is toxic in the cytosol but not in synaptic vesicles [1], [2], [37]. Consistently, we found that VMAT/CAT-1 knockout worms displayed slightly faster rates of DAergic neuron degeneration than controls (Figure 6A). If hαSyn-mediated altered dopamine metabolism contributes to hαSyn-mediated dopamine neuron degeneration, one would expect that *in vivo* overexpression of VMAT/CAT-1 would ameliorate hαSyn mediated DAergic neuron degeneration. Indeed, we found that VMAT/CAT-1 overexpression [34], [38] did prevent the hαSyn-mediated DAergic neuron degeneration (Figure 6B) and motor activity deficit (Figure 6C).

Critically, VMAT/CAT-1 overexpression prohibited hαSyn-mediated [DOPAC] upregulation (Figure 6E), but not [dopamine] upregulation (Figure 6D), providing evidence that enhanced sequestration of dopamine protects DAergic neurons from the toxicity of hαSyn expression by affecting dopamine turnover. Thus, hαSyn-mediated disruption of dopamine homeostasis significantly contributes to the observed DAergic neuron degeneration and loss of motor activity. Consistent with this conclusion, hαSyn expression disturbed the VMAT/CAT-1::GFP expression pattern in L2 organisms before significant DAergic neuron degeneration starts (Figures 4 and 6B), and this disruption persisted in the *cat-2* mutant background (Figure S5), wherein DAergic neuron degeneration was prevented.

## Discussion

Using *in vitro* and *ex vivo* mammalian or drosophila cell cultures, α-synuclein was found to disrupt dopamine homeostasis. Here, we provide *in vivo* evidence to support a critical relationship between α-synuclein and dopamine homeostasis. α-Synuclein may regulate dopamine homeostasis through multiple mechanisms [13], such as dopamine synthesis/breakdown [39], [40], compartmentalization [41] and recycling [42]. Consistently, we found that α-synuclein expression altered the expression of CAT-2/TH and distribution of dopamine synaptic vesicles.

Why did we observe an hαSyn mediated dopamine functional deficit along with upregulated dopamine synthesis and content? One possibility to explain this paradox is that hαSyn alters dopamine synaptic vesicle trafficking or packing, which may reduce the availability of dopamine synaptic vesicles at synapses and stimulate dopamine synthesis through feedback control mechanisms [43], [44]. Insufficient loading of unregulated dopamine into vesicles, therefore, could result in the observed altered dopamine metabolism. Indeed, α-synuclein was proposed to intervene directly in dopamine synaptic loading in mammals [12], [32], [33]. But this possibility should be further explored and validated with mammalian models.

In a previous study, investigators observed that heterological hαSyn expression in worm DAergic neurons induced dopamine deficiency rather than upregulation [21]. Interestingly, hαSyn expression did not cause degeneration of DAergic somas in their worm lines either. The less severe cytotoxicity of hαSyn in their worm line, compared with our hαSyn expressing lines and a line reported by Caldwell's group [22], may be due to different levels of protein expression.

It is worthy to point out that knockout of TH/CAT-2 or overexpression of VMAT/CAT-1 did not completely protect DAergic neurons from hαSyn-mediated degeneration. Consistently, the effect of knocking out VMAT/CAT-1 on DAergic degeneration was not as pronounced as that resulting from hαSyn expression, indicating that hαSyn-mediated cytotoxicity is not solely caused by the disruption of dopamine homeostasis. Indeed, α-synuclein mediated modification of chaperone-mediated autophagy (CMA) also plays a critical role in DAergic neuron loss in mammals [45].

## Materials and Methods

### *C. elegans* Strains

The promoter of *dat-1* was cloned and linked to a full-length cDNA encoding hαSyn, DsRed or GFP according to a previous description [21]. Transgenic lines expressing hαSyn were generated by injecting constructs of hαSyn (10 ng/µl per injection), DsRed or GFP under the control of the *dat-1* (encoding dopamine transporter) promoter sequence [46]. Two transgenic lines expressing hαSyn were obtained and both exhibited similar hαSyn toxicity. After the transgenic line expressing hαSyn was integrated, this integrated line was backcrossed 4× with wild type worms. To produce lines expressing both hαSyn and CAT-1::GFP or CAT-2::GFP, the transgenic hαSyn expressing worm line was crossed into nuls26 or EM641, respectively [24], [34]. All other worm protocols involved standard methods [47]. *cat-1* and *cat-2* mutants used were e1111 and e1112, respectively. N2 was used as the wild type.

### Immunochemistry

Worms were fixed with formaldehyde and stained with goat anti-hαSyn antibody according to published protocols with slight modifications [48]. All antibodies were purchased from Millipore.

### Microscopy

All confocal experiments were conducted with a Leica TCS SP2 confocal microscope. The spectra used were: DsRed(λ_ex_ = 543nm and λ_em_ = 580–630nm) and GFP(λ_ex_ = 488nm and λ_em_ = 510–530nm). To count fluorescent DAergic neuron numbers, living worms were immobilized with 30 mM sodium azide on 5% agarose pads and examined with a Leica DMI3000 microscope or a Leica TCS SP2 confocal microscope according to a published method with modifications [21]. Specifically, fluorescent DAergic neurons numbers were counted manually. The existence of a fluorescent DAergic soma was evaluated by its fluorescence intensity, its position in animals and the position of its dendrites of a candidate neuron. The position of a neuron in worms and the position of its dendrites are relatively unchanged in worms throughout their life [49]. To obtain consistent data, an observer was warmed up with 10–20 day 1 animals from an integrated wild type line expressing DsRed in DAergic neurons, every day when such an experiment was conducted. These animals have eight fluorescent DAergic somas. In these experiments, representative images were captured with an Andor iXon^EM^ 885 EMCCD camera and SimImaging (Feng, Z. unpublished software) (when Lecica DMI3000 microscope was used) or a Leica TCS SP2 confocal microscope. All images were processed and analyzed with National Instruments Vision Assistant 7.1.

### Behavioral Analyses

Worm basal/enhanced slowing responses with and without dopamine pretreatment were obtained as previously described [21], [25]. Locomotion speed was collected by using Automated and Quantitative Analysis of Behavior of Nematode (AQUABN) with a protocol described previously [26], [27]. After a 10-minute video was collected, the average speed from minutes 7–10 was computed to eliminate the locomotion acclimation phase. For dopamine rescue experiments, dopamine was added to the liquid medium before pouring Nematode Growth Medium (NGM) plates. Animals were then raised and experiments were conducted on dopamine containing NGM plates. These dopamine-exposed, hαSyn-expressing animals exhibited DAergic neurite and soma degeneration phenotypes similar to hαSyn expressing animals raised on regular NGM plates (data not shown).

### Dopamine and DOPAC Measurements

Samples were prepared as described [21] and filtered with a 0.45 µm Millipore filter before being injected into tandem LC-MS that employed an ESI probe in the positive ion mode. The column used was a C18 Discovery HS (5µm narrow bore), 15 cm long with a 2.1 mm diameter. The mobile phase used for elution was composed of solvent A (10 mM ammonium formate, pH 3.0) and solvent B (acetonitrile) with ratios ranging from 97% – 80% of solvent A. The detector was set up for single ion monitoring m/z 150–210.

### Statistical Analysis

Statistical significance was analyzed by using Statistica (StatSoft, Inc.). T-tests, ANOVA with Bonferroni corrections or Dunnet's post-hoc analyses were used for their appropriate applications.

## Supporting Information

Figure S1 Immunohistochemical analysis of hαSyn expression in transgenic *C. elegans*. A–D, Confocal images of a formaldehyde-fixed day 2 adult worm with DAergic neuron specific expression of hαSyn and DsRed. A, Bright field (BF). B, DsRed. C, hαSyn immunostaining (green). D. Merged image of B and C.
(0.80 MB TIF)

Figure S2 Immunohistochemical analysis of TH expression in transgenic *C. elegans*. A–B, Confocal images of formaldehyde-fixed day 0 (A) or day 10 (B) worm with DAergic neuron specific expression of hαSyn and DsRed. Left, TH immuostaining; Middle, DsRed; Right, Merged image of TH staining and DsRed; C, Quantification of DAergic neuron degeneration by using TH staining (green) or DsRed (Red). Data represents mean ± S.E.M., n = 10. 1 represents 6.8 and 6.3 DAergic neurons in DsRed and TH staining experiments, representatively.
(0.69 MB TIF)

Figure S3 Correlation of DAergic neuron degeneration with CAT-2::GFP and DsRed. A–B, Confocal images of living day 0 (A) or day 10 worms (B) with DAergic neuron specific expression of CAT-2::GFP, DsRed and hαSyn. Left, CAT-2::GFP; Middle, DsRed; Right, Merged image of CAT-2::GFP and DsRed. (C) Quantification of DAergic neuron degeneration by using CAT-2::GFP (green) or DsRed (red) in hαSyn-expressing (diamonds) and control (circles) lines. Data represent mean ± S.E.M., n = 30. Error bars may hide in symbols. ***, p<0.005 (Two-way ANOVA to compare hαSyn expressing and control line) (green: CAT-2::GFP; Red DsRed). 1 represents 7.9 and 7.7 in DsRed and CAT-2/TH::GFP experiments, respectively.
(1.44 MB TIF)

Figure S4 hαSyn expression does not affect serotonin neurotransmission. Enhanced slowing responses of day 2 adult worms. GFP indicates a wild type worm line expressing GFP in DAergic neurons. Food response experiments were conducted with (grey bars) or without (black bars) food.
(0.23 MB TIF)

Figure S5 hαSyn expression disrupts dopamine synaptic vesicle distribution in TH/CAT-2 knockout background. A–D, Typical bright field (A) or confocal laser scanning VMAT/CAT-1::GFP (B–D) images of living L2 worms expressing both VMAT/CAT-1::GFP and hαSyn in a TH/CAT-2 knockout background. C and D are magnified areas of B that show DAergic and serotonergic somas (C) or DAergic dendrites of CEPs (D), respectively. E, Quantification of CAT-1::GFP redistribution in CEPs of L2 worms expressing both VMAT/CAT-1::GFP and hαSyn in wild type (n = 5) or a TH/CAT-2 knockout background (n = 5). Error bar:SEM.
(0.98 MB TIF)
