# Supplementary figures and images for: Alpha-Synuclein Disrupted Dopamine Homeostasis Leads to Dopaminergic Neuron Degeneration in Caenorhabditis elegans
Source: PLoS One. 2010 Feb 19;5(2):e9312. doi: 10.1371/journal.pone.0009312 (PMC2824852; doi:10.1371/journal.pone.0009312)

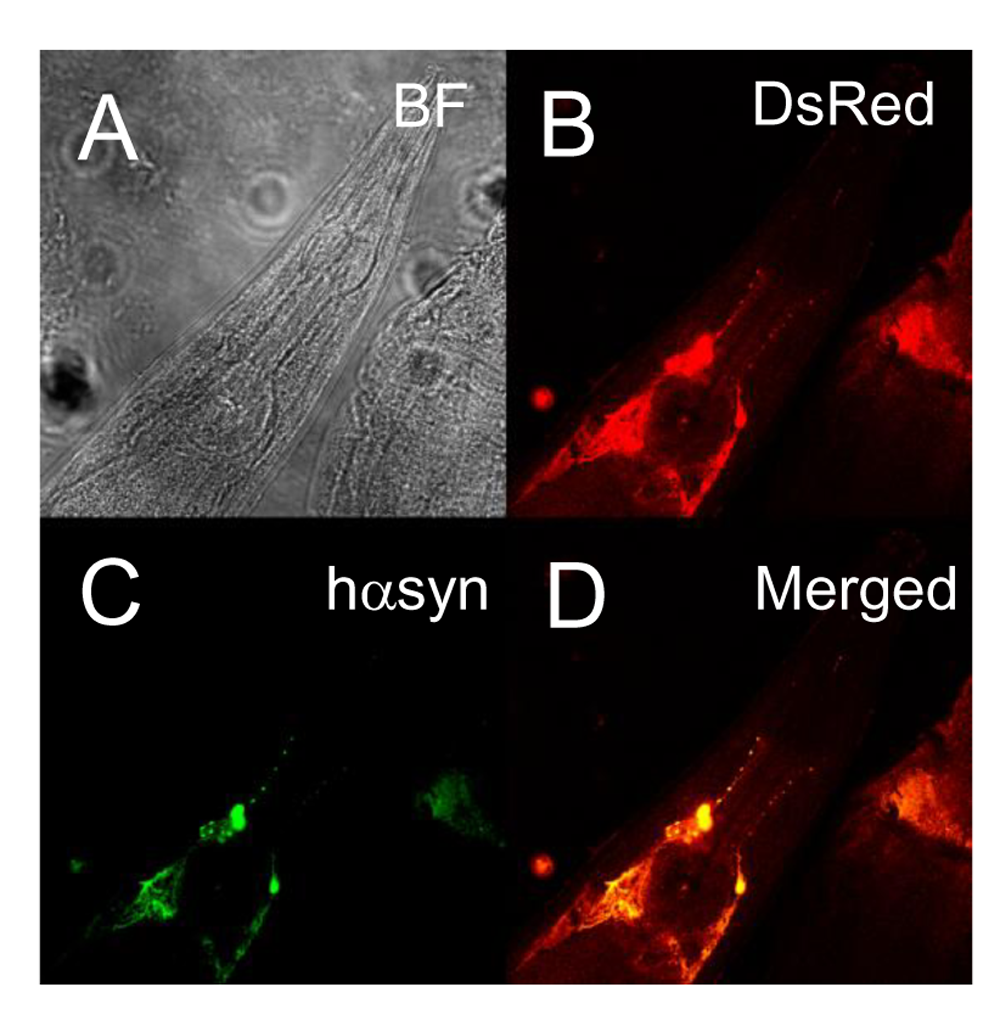

Supplement: Figure S1 — Immunohistochemical analysis of hαSyn expression in transgenic C. elegans. A–D, Confocal images of a formaldehyde-fixed day 2 adult worm with DAergic neuron specific expression of hαSyn and DsRed. A, Bright field (BF). B, DsRed. C, hαSyn immunostaining (green). D. Merged image of B and C. (0.80 MB TIF) [file pone.0009312.s001.tif]

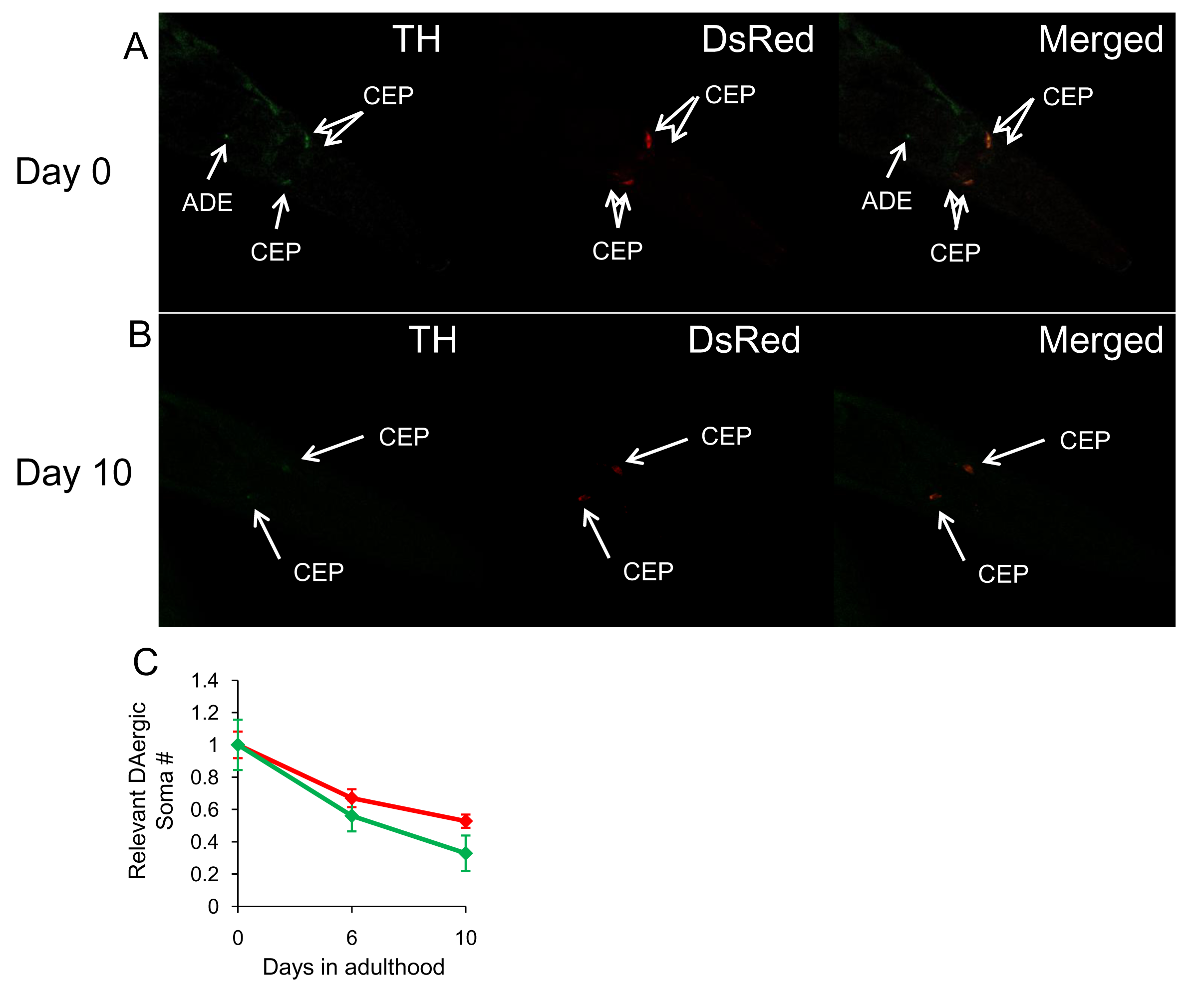

Supplement: Figure S2 — Immunohistochemical analysis of TH expression in transgenic C. elegans. A–B, Confocal images of formaldehyde-fixed day 0 (A) or day 10 (B) worm with DAergic neuron specific expression of hαSyn and DsRed. Left, TH immuostaining; Middle, DsRed; Right, Merged image of TH staining and DsRed; C, Quantification of DAergic neuron degeneration by using TH staining (green) or DsRed (Red). Data represents mean ± S.E.M., n = 10. 1 represents 6.8 and 6.3 DAergic neurons in DsRed and TH staining experiments, representatively. (0.69 MB TIF) [file pone.0009312.s002.tif]

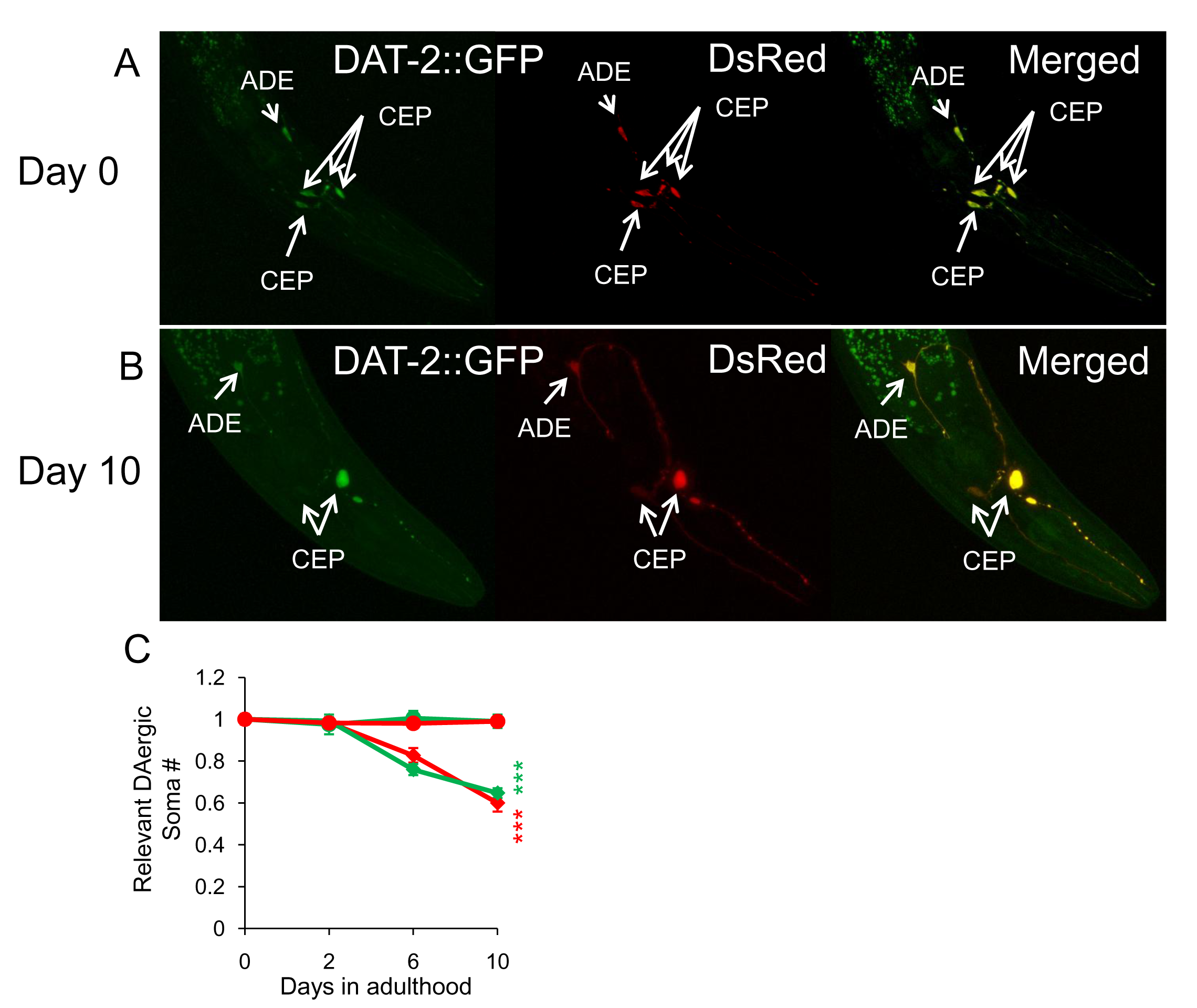

Supplement: Figure S3 — Correlation of DAergic neuron degeneration with CAT-2::GFP and DsRed. A–B, Confocal images of living day 0 (A) or day 10 worms (B) with DAergic neuron specific expression of CAT-2::GFP, DsRed and hαSyn. Left, CAT-2::GFP; Middle, DsRed; Right, Merged image of CAT-2::GFP and DsRed. (C) Quantification of DAergic neuron degeneration by using CAT-2::GFP (green) or DsRed (red) in hαSyn-expressing (diamonds) and control (circles) lines. Data represent mean ± S.E.M., n = 30. Error bars may hide in symbols. ***, p<0.005 (Two-way ANOVA to compare hαSyn expressing and control line) (green: CAT-2::GFP; Red DsRed). 1 represents 7.9 and 7.7 in DsRed and CAT-2/TH::GFP experiments, respectively. (1.44 MB TIF) [file pone.0009312.s003.tif]

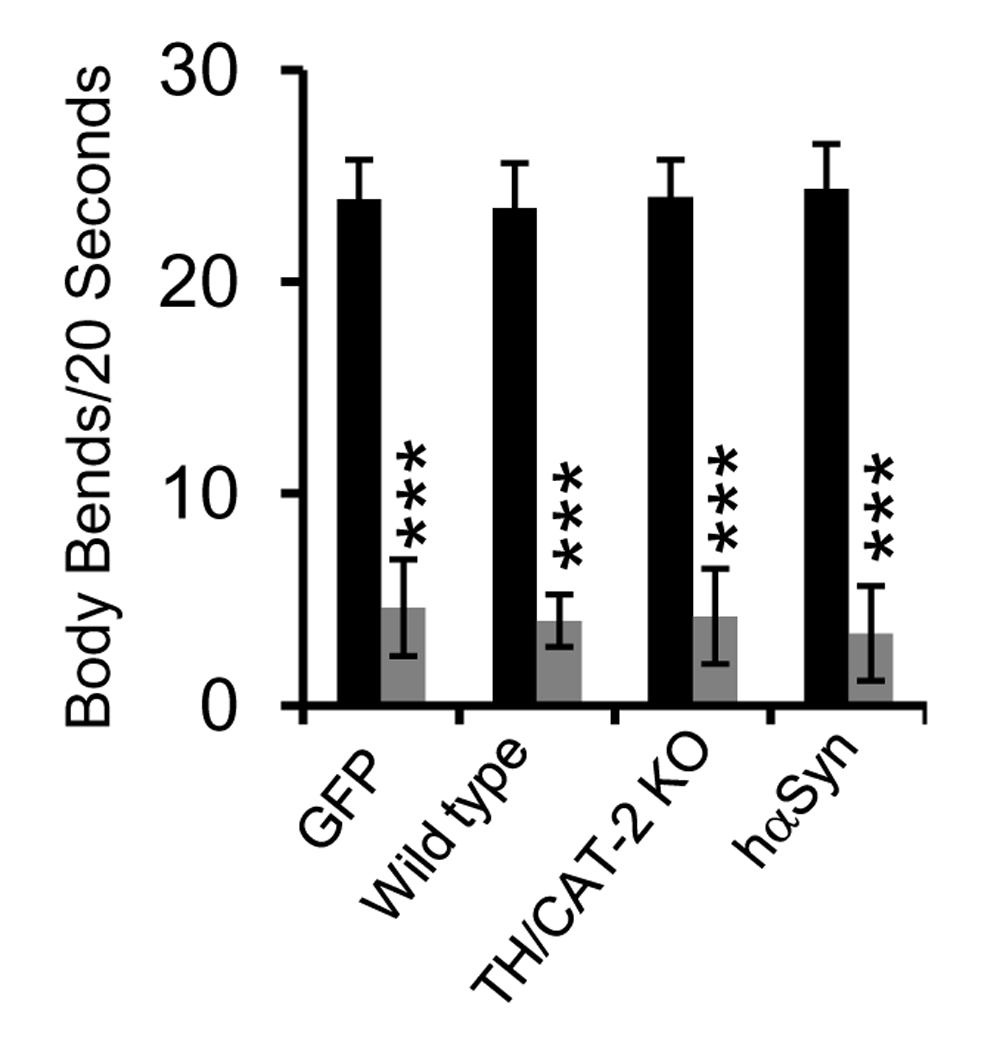

Supplement: Figure S4 — hαSyn expression does not affect serotonin neurotransmission. Enhanced slowing responses of day 2 adult worms. GFP indicates a wild type worm line expressing GFP in DAergic neurons. Food response experiments were conducted with (grey bars) or without (black bars) food. (0.23 MB TIF) [file pone.0009312.s004.tif]

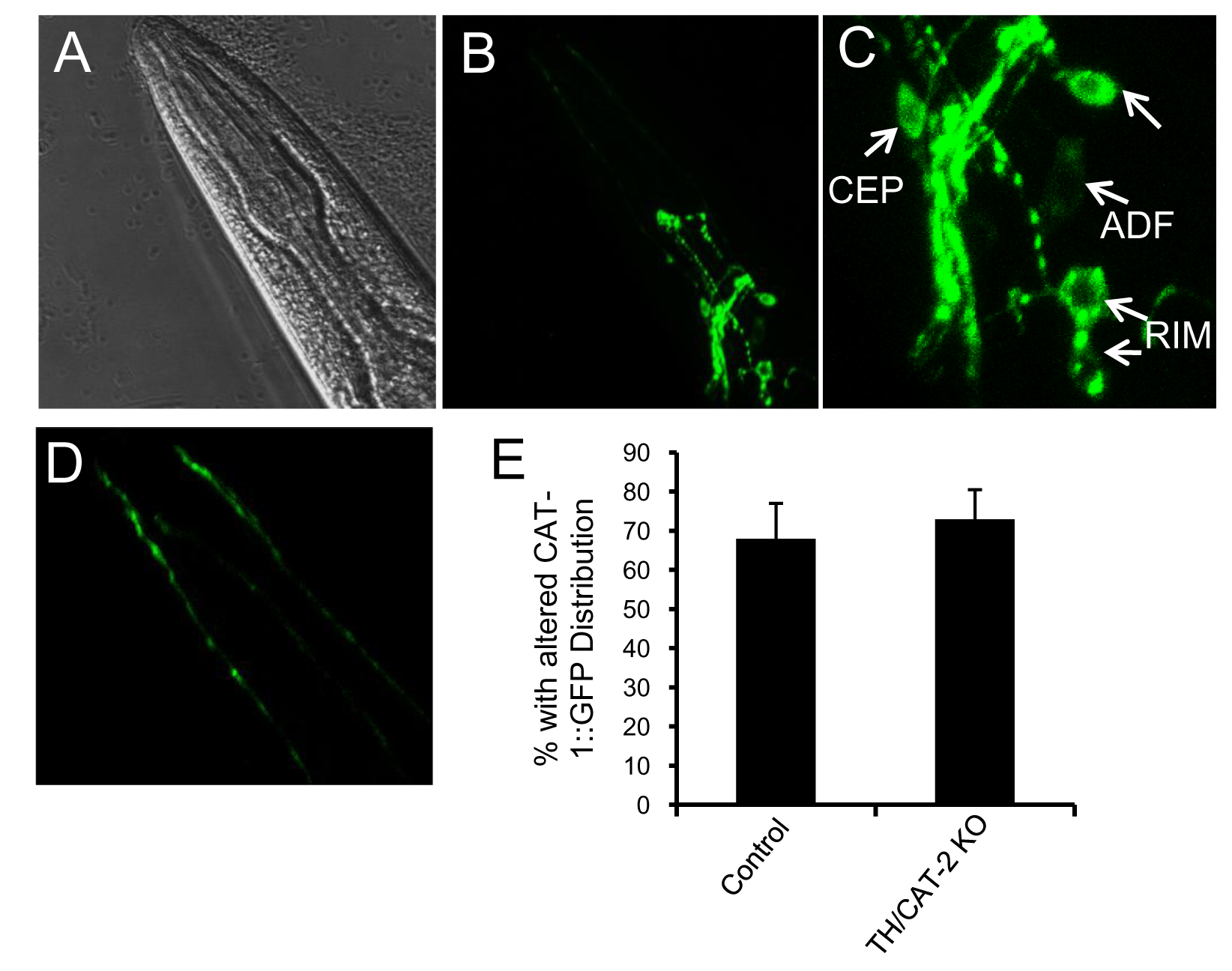

Supplement: Figure S5 — hαSyn expression disrupts dopamine synaptic vesicle distribution in TH/CAT-2 knockout background. A–D, Typical bright field (A) or confocal laser scanning VMAT/CAT-1::GFP (B–D) images of living L2 worms expressing both VMAT/CAT-1::GFP and hαSyn in a TH/CAT-2 knockout background. C and D are magnified areas of B that show DAergic and serotonergic somas (C) or DAergic dendrites of CEPs (D), respectively. E, Quantification of CAT-1::GFP redistribution in CEPs of L2 worms expressing both VMAT/CAT-1::GFP and hαSyn in wild type (n = 5) or a TH/CAT-2 knockout background (n = 5). Error bar:SEM. (0.98 MB TIF) [file pone.0009312.s005.tif]
